# Population Genomics of Japanese Macaques (*Macaca fuscata*): Insights Into Deep Population Divergence and Multiple Merging Histories

**DOI:** 10.1093/gbe/evaf001

**Published:** 2025-01-07

**Authors:** Atsunori Higashino, Katsuki Nakamura, Naoki Osada

**Affiliations:** Center for the Evolutionary Origins of Human Behavior, Kyoto University, Inuyama, Aichi 484-8506, Japan; Center for the Evolutionary Origins of Human Behavior, Kyoto University, Inuyama, Aichi 484-8506, Japan; Faculty of Information Science and Technology, Hokkaido University, Sapporo, Hokkaido 060-0814, Japan

**Keywords:** Japanese macaques, genetic diversity, population dynamics

## Abstract

The influence of long-term climatic changes such as glacial cycles on the history of living organisms has been a subject of research for decades, but the detailed population dynamics during the environmental fluctuations and their effects on genetic diversity and genetic load are not well understood on a genome-wide scale. The Japanese macaque (*Macaca fuscata*) is a unique primate adapted to the cold environments of the Japanese archipelago. Despite the past intensive research for the Japanese macaque population genetics, the genetic background of Japanese macaques at the whole-genome level has been limited to a few individuals, and the comprehensive demographic history and genetic differentiation of Japanese macaques have been underexplored. We conducted whole-genome sequencing of 64 Japanese macaque individuals from 5 different regions, revealing significant genetic differentiation and functional variant diversity across populations. In particular, Japanese macaques have low genetic diversity and harbor many shared and population-specific gene loss, which might contribute to population-specific phenotypes. Our estimation of population demography using phased haplotypes suggested that, after the strong population bottleneck shared among all populations around 400 to 500 kya, the divergence among populations initiated around 150 to 200 kya, but there has been the time with strong gene flow between some populations after the split, indicating multiple population split and merge events probably due to habitat fragmentation and fusion during glacial cycles. These findings not only present a complex population history of Japanese macaques but also enhance their value as research models, particularly in neuroscience and behavioral studies. This comprehensive genomic analysis sheds light on the adaptation and evolution of Japanese macaques, contributing valuable insights to both evolutionary biology and biomedical research.

**Significance**

In this study, we analyze high-coverage whole-genome data of 64 Japanese macaque (*Macaca fuscata*) individuals across the Japanese archipelago to elucidate their population history and its genomic consequences. We revealed strong genetic differentiation among local populations with considerably small effective population sizes, and each population harbors a number of characteristic loss-of-function mutations affecting hundreds of genes, a part of which may contribute to the population-specific phenotypes of this species. The initial divergence among populations was estimated to have occurred around 150 to 200 kya based on cross species coalescent analysis, which further suggested the subsequent glacial cycles served as factors for both merging and splitting of local populations, highlighting the importance of environmental fluctuation for shaping the pattern of genomic diversity in nonhuman primates.

## Introduction

The influence of long-term climatic changes on the history of living organisms has been a subject of research for decades (e.g. Willis et al. 2004; Cooper et al. 2015). Glacial cycles are among the most significant factors, having extensively altered global species distributions and impacted population sizes (Haffer 1969; Provan and Bennett 2008). However, the detailed population dynamics during these glacial cycles and their effects on genetic diversity and genetic load have been explored on a genome-wide scale in a limited number of organisms (e.g. Lorenzana et al. 2022; Lozier et al. 2023; Feng et al. 2024). Recent studies in population genomics have unveiled that actual population histories are far more complex than previously thought, often involving multiple splits and merges, as suggested by research on archaic and modern humans (Bergström et al. 2020; Hubisz et al. 2020; Ragsdale et al. 2023). Studying species that have endured harsh environments over extended periods, particularly those forming multiple structured populations, can offer valuable insights into how environmental changes have shaped genomic diversity of species.

The Japanese macaque (*Macaca fuscata*), a species of the family Cercopithecidae, endemic to the Japanese archipelago, belongs to the *mulatta* group within the genus *Macaca* (Zinner et al. 2013). The *mulatta* group consists of three species: *M. mulatta*, *M. cyclopis*, and *M. fuscata*, with *M. fuscata* being the most distantly related to the other two. The divergence time of *M. fuscata* to the others has been estimated to be around 1.0 to 2.3 Ma using whole-genome datasets (Kuderna et al. 2023; Zhang et al. 2023; Zhou et al. 2024). However, Osada et al. (2021) demonstrated that *M. fuscata* is more closely related to Chinese *M. mulatta* than to Indian *M. mulatta*, suggesting past gene flow between *M. fuscata* and Chinese *M. mulatta* after the divergence of Chinese and Indian *M. mulatta* that was estimated to have occurred around 162 kya (Hernandez et al. 2007).

As the nonhuman primate inhabiting the northernmost regions, the Japanese macaque has uniquely adapted to cold environments, having large body size, long fur, and extremely short tail. They distribute wide range of the Japanese archipelago except for the Hokkaido Island and the Ryukyu Islands. A population in the Yaku Islands, which locates the southernmost area Japanese macaques inhabiting, is often categorized as a different subspecies, *M. fuscata yakui*, although genetic studies using nuclear genome markers rejected the monophyly of *M. f. yakui* (Ito et al. 2021). Fossil records suggested that they have inhabited in the Japanese archipelago at least 430 kya, but their evolutionary history is poorly understood (Aimi 2002). Studies in population genetics have extensively explored the lineage of Japanese macaques. Analyses comparing mitochondrial DNA haplotypes reveal genetic differentiation between the Western and Eastern populations, with the Eastern populations exhibiting lower nucleotide diversity than their Western counterparts (Kawamoto et al. 2007). Furthermore, among the Eastern populations, the Shimokita Peninsula population exhibits lower genetic diversity, attributed to the restricted gene flow with neighboring populations (Kawamoto et al. 2008). Ito et al. (2021) performed double-digested restriction-site associated DNA sequencing (ddRAD-seq) including a wide range of Japanese macaque samples across the Japanese archipelago and clarified their fine-scale genetic structure (Ito et al. 2021). They also performed ecological niche modeling and showed that their habitat was severely restricted to south coast of Japanese archipelago during the Last Glacial Maximum (LGM). The ddRAD-seq analysis by Ito et al. (2021) and other phylogeographic studies based on mitochondrial DNA sequences (Kawamoto et al. 2007; Kawamoto 2010) provided a general overview of genetic structure of Japanese macaques; however, remaining questions including a fine-scale demographic trajectories over time and genomic diversity at the nucleotide level have not thoroughly investigated.

The Japanese macaques have also been used as experimental animals, particularly by Japanese researchers for physiological and behavioral studies. In the field of neuroscience, Japanese macaques are particularly valued and employed for their intelligence, docility, and adeptness with their hands. The Japanese government hosts the National BioResource Project (NBRP), funded by the Ministry of Education, Culture, Sports, Science and Technology, providing a repository of Japanese macaques for research purposes (Isa et al. 2009). The NBRP for Japanese macaques has strategically introduced macaques from various regions across Japan, establishing breeding populations that preserve genetic diversity. Research into these populations has uncovered phenotypic variations unique to local groups of Japanese macaques. Over 10 years of daily breeding observations have highlighted the distinct characteristics inherent to each local group within the NBRP colonies. For instance, the Minoh population exhibits a tendency toward obesity, the Hagachizaki population shows a remarkable lack of fear toward humans, and the Okazaki population is notable for being free from Simian T-cell leukemia virus infection. While these populations from diverse geographical regions of Japanese archipelago have been maintained separately, showing unique phenotypic traits, the extent of their genetic differentiation at a genome-wide level has yet to be fully understood. A deeper understanding of their genetic backgrounds could significantly enhance the utility of Japanese macaques as valuable resources for scientific research.

Compared with other species in the *mulatta* group, the genomic information of Japanese macaques is quite limited, and only a few individual genomes have been fully sequenced (Osada et al. 2021). Here, we performed whole-genome sequencing of 64 Japanese macaque individuals originated from 5 geographic locations and reared in NBRP and clarify their genomic background, particularly focusing on how many neutral and potentially functional variants were carried in the populations. We also sequenced four trios from different populations and performed haplotype phasing using the information. This large-scale dataset and phased haplotype panels would become important genetic resources for future experimental and genetic studies using Japanese macaques. We found the level of genetic differentiation among populations is quite high and highly differentiated variants caused the functional loss of hundreds of genes in each population, which potentially contribute to characteristic phenotypes in each population. The population history of Japanese macaques was also inferred from the whole-genome dataset, showing a highly complex history.

## Results

### Genome Sequencing of Japanese Macaques From Five Populations

**Fig. 1.**
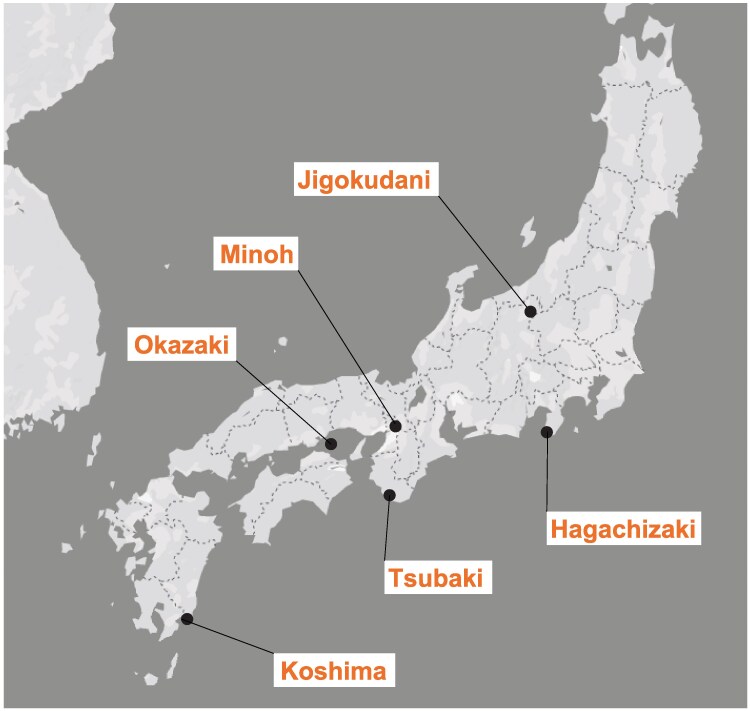
Locations of Japanese macaque samples analyzed in this study. The locations include the following: Jigokudani (Yamanouchi Town, Nagano Prefecture); Hagachizaki (Minami-Izu Town, Shizuoka Prefecture); Minoh (Minoh City, Osaka Prefecture); Tsubaki (Shirahama Town, Wakayama Prefecture); Okazaki (Shodo-shima Town, Kagawa Prefecture); and Koshima (Kushima City, Miyazaki Prefecture).

We sequenced the genomes of 64 Japanese macaques from 5 NBRP colonies—Jigokudani, Hagachizaki, Minoh, Tsubaki, and Okazaki—with their geographical origins presented in Fig. 1 with details in supplementary table S1, Supplementary Material online. Although reared in captivity, all sequenced samples originated as founders from wild populations. Additionally, genomic data for one Koshima individual, seven Chinese rhesus macaques (RMC) (*M. mulatta*), two Indian rhesus macaques (RMI), one Taiwanese macaque (*M. cyclopis*), and one Thai cynomolgus macaque (CMT) (*M. fascicularis*) were retrieved from the public databases (supplementary table S2, Supplementary Material online) (Xue et al. 2016; Liu et al. 2018; Osada et al. 2021). Using the rhesus macaque reference genome (Mmul_10), we mapped short reads to identify single nucleotide variants (SNVs) and short indels. After filtering, a total of 20,888,045 SNVs and 5,581,784 short indels were detected on the autosomes of Japanese macaque samples. Of these, 4,055,738 SNVs were fixed among all Japanese macaque individuals. On the X chromosome, we identified 543,735 SNVs and 197,404 indels.

**Fig. 2.**
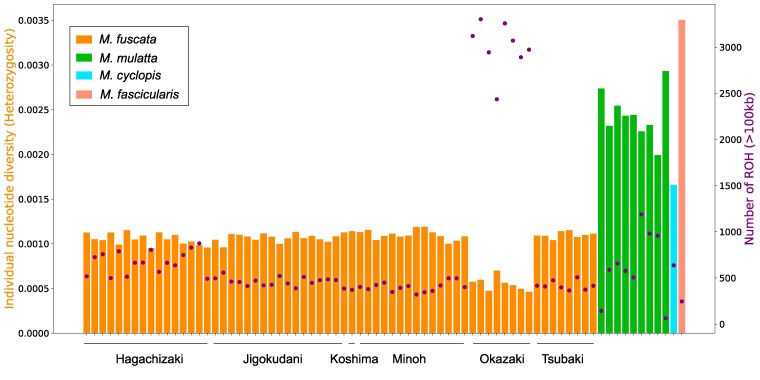
Individual nucleotide diversity (heterozygosity) and the number of ROH segments >100 kb. Bar heights represent individual nucleotide diversity (left *y* axis), while circles indicate the number of ROH segments (right *y* axis).

For each Japanese macaque individual, we detected 4,000 to 10,000 heterozygous SNVs among 1,930,114,258 autosomal sites that passed our filtering criteria. We evaluated individual nucleotide diversity (heterozygosity per base pair), representing the nucleotide difference per site between two autosomes for each individual. The values were ∼0.08% to 0.10%, with the notable exception of the Okazaki population, which exhibited extremely lower heterozygosity at around 0.04%. Compared with the Japanese macaques, other macaque species within the *mulatta* group showed considerably higher heterozygosity: 0.14% in the Taiwanese macaque and 0.16% to 0.24% in rhesus macaques (Fig. 2). Grouping all unrelated Japanese individuals and calculating the nucleotide diversity among all Japanese macaques yield a value of 0.13%, which still remains lower than those observed in other macaque samples.

We also assessed the number and length of runs of homozygosity (ROH) in each genome. These two values showed a negative correlation with individual nucleotide diversity. Notably, individuals in the Okazaki population exhibited a strikingly higher number and longer ROHs compared with Japanese macaques from other populations (Fig. 2; supplementary table S3, Supplementary Material online).

### Functional Impact of Variants in Japanese Macaques

The low genetic diversity in Japanese macaques suggests they underwent significant population bottlenecks, likely during their migration to the Japanese archipelago or habitat reductions in glacial periods. We focused on functional mutations universally present in the autosomes of Japanese macaque populations. In total, the Japanese macaques exhibit 12,907 fixed nonsynonymous substitutions against the rhesus macaque reference genome, some of which may be shared with other macaque species. In order to focus on Japanese macaque-specific derived variants with functional importance, we only considered the sites where all 11 outgroup individuals, *M. fascicularis, M. mulatta*, and *M. cyclopis* (supplementary table S2, Supplementary Material online), unambiguously have the reference allele as the homozygous state. We identified 40 SNVs and 53 short indels resulting in loss-of-function (LoF), caused by frameshifts, premature stop codons, and splicing disruptions, fixed and specific to the Japanese macaque samples.

On average, each Japanese macaque carries 39.6 and 84.0 species-specific homozygous LoF variants segregating within the species due to SNVs and indels, respectively, in addition to the fixed LoF substitutions. Including heterozygous variants, Japanese macaque individuals on average have 828 genes with species-specific LoF variants, compared with the rhesus macaque reference genome. Although individuals in the Okazaki population had a higher number of homozygous LoF variants, the total number of genes affected by LoF was significantly lower in this population compared with the others (*P* = 5.52 × 10^−6^, two-sided Mann–Whitney *U* test).

### Highly Differentiated Variants Among Japanese Macaque Populations

To elucidate the genetic underpinnings of population differentiation, we assessed the *F*_ST_ between populations. *F*_ST_ values on autosomes between pairwise populations were notably high, spanning from 0.112 to 0.416 across autosomes, with the Okazaki population displaying the highest *F*_ST_ when compared with others (Table 1). Notably, *F*_ST_ values on the X chromosomes consistently exceeded those on autosomes, ranging from 0.206 to 0.580. We further evaluated the *Q* statistics, serving as a relative impact of genetic drift on autosomes versus X chromosomes (Keinan et al. 2009). Under conditions of equal population genetics parameters, such as effective population size and migration rate for both sexes, this value is anticipated to be 0.75. Excluding the highly differentiated Okazaki population, for which *Q* statistics computation was impractical, *Q* values were between 0.36 and 0.50.

**Table 1:** Pairwise *F*_ST_ between populations

	Hagachizaki	Jigokudani	Minoh	Tsubaki	Okazaki
Hagachizaki	…	0.112 (0.001)	0.200 (0.001)	0.209 (0.001)	0.416 (0.003)
Jigokudani	0.206 (0.011)	…	0.198 (0.001)	0.209 (0.002)	0.415 (0.003)
Minoh	0.371 (0.013)	0.369 (0.014)	…	0.137 (0.002)	0.336 (0.003)
Tsubaki	0.369 (0.011)	0.370 (0.009)	0.252 (0.011)	…	0.368 (0.003)
Okazaki	0.580 (0.015)	0.580 (0.015)	0.447 (0.020)	0.500 (0.012)	…

Upper right and lower left triangles show the *F*_ST_ values on autosomes and X chromosomes, respectively. Standard errors are presented in the parentheses.

We subsequently concentrated on highly differentiated variants leading to LoF, which is likely to contribute population-specific phenotypes. To identify population-specific variants, we calculated population-specific *F*_ST_ within Japanese macaques for each biallelic SNV and short indel. We also focused on Japanese macaque-specific derived SNVs, following the approach used in the LoF analysis. The analysis compared allele frequencies in each target population with those in the background populations of Japanese macaques, excluding individuals within the third degree of kinship. Variants falling within the top 1% of *F*_ST_ values were operationally designated as population-specific SNVs although they are not necessarily exclusively found in the target population. From these, we selected population-specific LoF variants for each population. We discovered that each population possesses population-specific LoF variants, affecting 34 to 48 genes. We performed functional enrichment analysis using DAVID for these genes. The genes associated with population-specific LoF variants in the Minoh and Tsubaki populations were initially found to be significantly enriched for functions related to Cytochrome Oxidase P450 genes. However, we found that this excess was due to the misassignment of ENSEMBL Gene IDs to gene symbols in the DAVID database. A comprehensive list of population-specific LoF genes is available in supplementary table S4, Supplementary Material online.

To verify the existence of population-specific LoF variants that might be associated with unique phenotypic traits in each population, we chose three genes harboring population-specific LoF variants for closer examination through Sanger sequencing. These genes included *TECR*, associated with neuronal disorders in humans (Çalışkan et al. 2011), found in the Hagachizaki population, and *ISG20L2* and *CTSW*, both related to immune functions, identified in the Okazaki population. For each of these three genes, we were able to confirm that the LoF variants occur at high frequencies within their respective populations. Possible phenotypic effects of these mutations are discussed in the “Discussion” section.

### Population Structure of Japanese Macaques

**Fig. 3.**
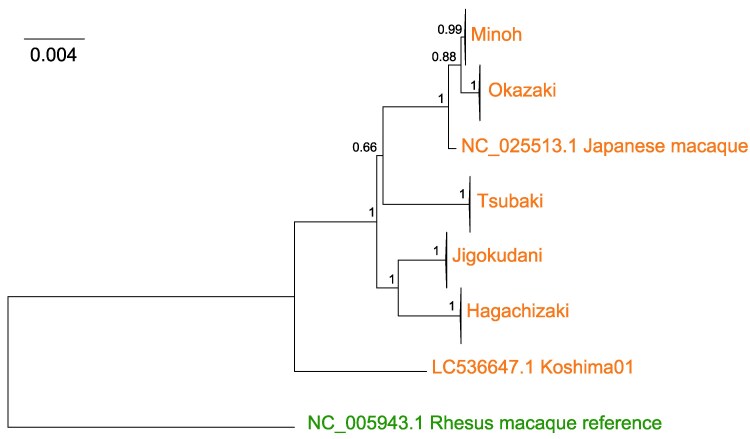
Phylogenetic tree of mitochondrial genomes. Bootstrap values are shown upon the nodes. Samples in populations are collapsed because all samples in the same population were tightly clustered.

We inferred population history of Japanese macaques using various genetic data. The whole mitochondrial genome sequences were initially assembled from the short-read data, and phylogenetic tree was reconstructed. The genealogy of mitochondrial genomes, including outgroup species, is shown in Fig. 3. In the mitochondrial tree, among Japanese macaques, the Koshima individual split first, and then Jigokudani–Hagachizaki and Minoh–Tsubaki–Okazaki split. Although the Okazaki population is highly differentiated from the other populations in the analysis of genome-wide *F*_ST_, the mitochondrial genomes of Okazaki population is highly similar to those of Minoh population.

**Fig. 4.**
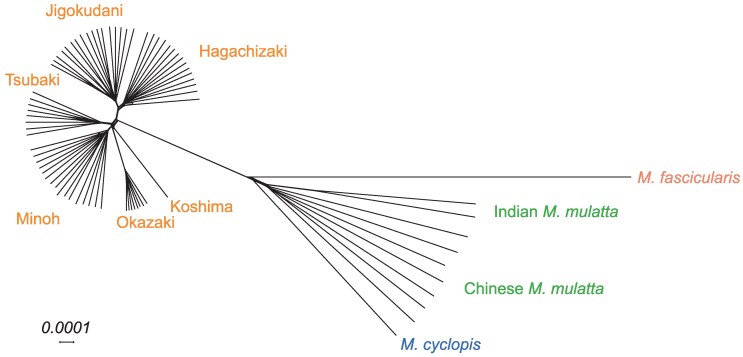
The Neighbor-Net graph, constructed from nuclear genome data. The genetic distances were calculated for every pair of samples, and the network was subsequently generated using SplitTree software.

A Neighbor-Net graph constructed from the nuclear genome data is presented in Fig. 4. Although each population formed a single cluster, the pattern was incongruent with the mitochondrial tree. In the nuclear genome network, the Okazaki population was not genetically very close to the Minoh population. In addition, the Koshima individual, which had highly divergent mitochondrial haplotype, showed complex admixture pattern with the other populations.

We also performed principal component analysis (PCA) and admixture analysis for the Japanese macaque populations. In the PCA plot shown in supplementary fig. S1, Supplementary Material online, the PC1 scores correspond to the difference between the north-east and south-west groups. PC2 scores split the Okazaki population from the Minoh–Tsubaki–Koshima group. The PC3 scores isolate the Koshima individual. The scores of PC4 and PC5 represent the genetic differentiation between Minoh and Tsubaki and between Jigokudani and Hagachizaki, respectively. The admixture plot from *K* = 2 to 5 is shown in supplementary fig. S2, Supplementary Material online, where the cross-validation errors were the smallest when *K* = 3 (supplementary fig. S3, Supplementary Material online). The assignment of ancestry was generally in good agreement with the results of Neighbor-Net graph and PCA plot.

### Demographic Modeling

**Fig. 5.**
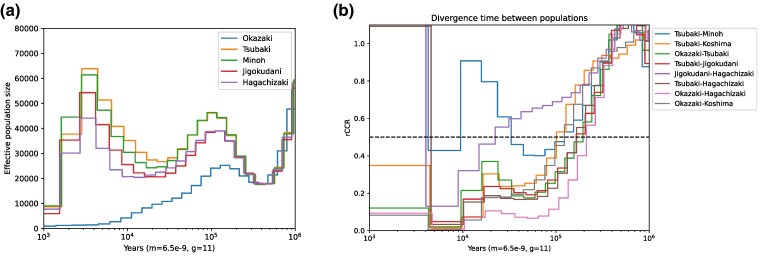
Coalescent rates over time in Japanese macaques. a) The graph illustrates the changes in effective population size over time for five Japanese macaque populations on the *y* axis, analyzed through eight phased haplotypes (equivalent to four individuals) per population. b) The graph presents the relative cross coalescent rates rCCRs among these populations on the *y* axis. In both panels, the *x* axis indicates time from the present, displayed on a log scale.

We inferred past population size changes of Japanese macaques using a coalescent framework implemented in MSMC2 software for each population (Schiffels and Wang 2020). The trajectories of population size change for five populations, assuming the mutation rate of 6.5 × 10^−9^ per generation and the generation time of 11 years (Itoigawa et al. 1992; Koyama, et al. 1992; Wang et al. 2020), are shown in Fig. 5a. The results suggested that all five populations experienced strong population bottleneck around 400 to 500 kya, After the bottleneck, all populations were inferred to have increased in size, reaching their peak around 100 to 150 kya. The population size of Okazaki appeared to have been decreasing until very recent, but those of other four populations increased again and became maximum around 3 to 4 kya.

The relative cross coalescent rates (rCCRs) are also estimated and presented in Fig. 5b. The pattern shows complex pattern of their divergence. Assuming that rCCR of 0.5 is the divergence time of populations, many population pairs, except for between Jigokudani and Hagachizaki populations, diverged around 150 to 200 kya. The rCCR between the Tsubaki and Minoh populations decreased around the time but started to increase after the dip of rCCR, suggesting the scenario in which these populations have partially merged after the initial divergence. The rCCR between the Jigokudani and Hagachizaki implied that they diverged around 20 to 30 kya, suggesting that they have not diverged until LGM. The rCCR between some pairs increased again after 3 kya. In any population pairs, the rCCR did not monotonically decrease, which appears to indicate that the populations were split and merged multiple times after the initial colonization to the Japanese archipelago.

### Genetic Relatedness Among Japanese Macaque Populations

The Neighbor-Net graph and population size trajectories imply complex population history of Japanese macaques. There is a general agreement of the division between Jigokudani–Hagachizaki and Minoh–Tsubaki groups. However, if we calculated *f*_4_ statistics and evaluated the relationship of quartets inferred by the Neighbor-Net graph, the value of *f*_4_(Jigokudani, Hagachizaki; Minoh, Tsubaki) was significantly deviated from 0 toward the positive direction (*Z*-score = 3.688). All other quartet topologies showed a significant deviation in *f*_4_ statistics. We next estimated the *f*_4_ statistics using the RMI as outgroup to test the symmetric sister relationships between Jigokudani and Hagachizaki, *f*_4_(RMI, Minoh/Tsubaki; Jigokudani, Hagachizaki) and between Minoh and Tsubaki, *f*_4_(RMI, Jigokudani/Hagachizaki; Minoh, Tsubaki). The *f*_4_ statistics showed bias in all configurations, but greater magnitude of gene flow was suggested for between Jigokudani and Minoh populations and between Hagachizaki and Tsubaki populations (supplementary table S5, Supplementary Material online).

The placement of Okazaki and Koshima populations is unclear from the Neighbor-Net graph. The outgroup *f*_3_ statistics using the RMI at outgroup showed that Koshima individual is most closely related to Okazaki population, and the Okazaki population is most closely related to the Minoh population (supplementary table S6, Supplementary Material online), which is consistent with the pattern observed in the Neighbor-Net graph (Fig. 3).

We also calculated the *f*_4_ statistics to assess the relatedness of Japanese macaques to RMC. Using *M. fascicularis* (CMT) as an outgroup, we computed *f*_4_(CMT, *X*; RMC, RMI), where *X* represents different Japanese macaque populations. All Japanese macaque populations exhibited strongly negative *f*_4_ values (supplementary table S7, Supplementary Material online), suggesting ancient gene flow between RMC and Japanese macaque lineages (Osada et al. 2021). We then tested the heterogeneity in the relatedness to RMC among Japanese macaque populations by calculating *f*_4_(RMI, RMC; *Y*, *Z*), where *Y* and *Z* represent all possible pairs of Japanese macaque populations. (supplementary table S8, Supplementary Material online). The results showed that Koshima individual is more distantly related to RMC compared with the other Japanese macaques.

## Discussion

In this study, we determined high-coverage whole-genome sequences of 64 Japanese macaque individuals derived from 5 population sources reared in NBRP. These samples include four trios, and we performed haplotype phasing using the information. The information provides valuable insight into genetic diversity of Japanese macaques.

### Low Genetic Diversity of Japanese Macaques

The entire samples sequenced in this study are wild-derived animals: i.e. founders of colonies maintained in NBRP. Although they have been fed by humans for a few decades and some samples were identified as closely related, we showed that genome-wide individual nucleotide diversity was almost constant within populations, indicating that they were not strongly inbred individuals. The genome-wide individual nucleotide diversity is around 0.1%, showing that Japanese macaques harbor similar genetic diversity with the human population and the values are much smaller than other species in the *mulatta* group macaques (Fig. 2).

The nucleotide diversity in the Okazaki population was around a half of that in the other Japanese macaques. The population size trajectory of Okazaki population presented in Fig. 5a shows that the decline of population size of the Okazaki population has probably continued since 100 to 150 kya, indicating that the low genetic diversity of the Okazaki population is not simply due to recent inbreeding or population bottleneck. Notably, the nucleotide diversity of the Okazaki population is one of the smallest values among wild primates (Osada 2015). Individuals in the Okazaki population also possessed extensive numbers and sizes of ROH, probably owing to recent population size reduction. However, we found that the number of genes affected by LoF variants was smaller in the Okazaki population than in the other populations. This is because individuals in the Okazaki population have fewer heterozygous LoF variants on average, suggesting that strongly deleterious LoF variants have already been purged to some extent in this population.

The divergence time of Japanese macaque lineages from the most recent common ancestors with rhesus macaques was estimated to be around 1.0 to 2.3 Ma using a whole-genome sequence data (Kuderna et al. 2023; Zhang et al. 2023; Zhou et al. 2024). All Japanese macaque populations shared the signature of strong population bottleneck around 400 to 500 kya. Since the oldest fossil of Japanese macaques was found in a layer dated around 430 to 630 kya (Aimi 2002), the bottleneck may correspond to the founding event of the colonization of Japanese macaques to the Japanese archipelago. The strong population bottleneck and long history of partial isolation would have increased the genetic load in Japanese macaques. Each Japanese macaque individual carries ∼120 species-specific unfixed LoF variants as homozygotes. Given that Icelandic humans, which have also experienced strong population bottleneck during migration, harbor on average 21 homozygous LoF variants including SNVs and indels (Gudbjartsson et al. 2015), the number of LoF variants carried by Japanese macaques are substantial, propounding significant impact on their phenotypes.

### Population Differentiation Among Japanese Macaques

Population differentiation among the Japanese macaque populations, as measured by *F*_ST_, was relatively high, particularly given that these macaques are restricted to the Japanese archipelago. This elevated *F*_ST_ is partly attributed to the small effective population size of each group and a history of isolation. The least differentiated pair, Hagachizaki and Jigokudani, showed an *F*_ST_ value of 0.112, comparable with the differentiation observed among African, European, and Asian human populations (Bhatia et al. 2013). The high level of differentiation observed in the Okazaki population is similar to that reported in golden snub-nosed monkeys (*Rhinopithecus roxellana*), an endangered species living in fragmented inland habitats (Zhou et al. 2016).

The *Q* statistic, representing the relative impact of genetic drift on autosomes vs X chromosomes (Keinan et al. 2009), was below 0.75 in all pairwise comparisons, except for those where the statistic could not be calculated due to high *F*_ST_. This pattern suggests a scenario in which males migrated more frequently than females between populations, consistent with observations of female philopatry in Japanese macaques (Sprague et al. 1998).

### Functional Differentiation in Proteins

Although many genes were specifically lost in a population, we investigated more deeply a part of candidate genes that would be the basis of population-specific phenotypes. We focused on behavior-related genes in the Hagachizaki population and immune system–related genes in the Okazaki population, as these traits are characteristic features of the colonies. It should be noted that these genes were arbitrarily selected for validation of our short-read sequencing, and many other candidate genes were also identified (supplementary table S3, Supplementary Material online). The Hagachizaki-specific LoF mutation in *TECR* (ENSMMUT00000055868.2) is a substitution from C to T, changing the codon for glutamine to stop codon. The premature stop codon reduces the length of intact protein from 374 to 262 aa long. *TECR*, a membrane protein involved in fatty acid elongation, is associated with mental retardation in humans (Çalışkan et al. 2011). This gene would be an intriguing candidate for characterizing the phenotypes of the Hagachizaki population, given their lack of fear toward humans. However, according to the UniProt database, the C-terminal region of protein (A0A1D5QQQ1) does not form rigid structure, and the mutation only affects the amino acid sequences of one transcript variant among three possible variants. In addition, the stop codon–gained transcript isoform was only annotated in the Ensembl database and not in the GenBank database, of *M. mulatta*, and it may result from an error in the database annotation. The mutation is almost exclusively found in the Hagachizaki population. Among 15 individuals we sequenced, 3 and 11 individuals were homozygous and heterozygous for the mutation, respectively. Only one individual in the Hagachizaki population was a complete wild type. On the other hand, only one individual in the Minoh population carries the mutation as heterozygous state in our dataset.

The frameshift-causing deletions in the *CTSW* (ENSMMUG00000008649) and *ISG20L2* (ENSMMUG00000000037) genes were found exclusively in the Okazaki population. Within this population, only one individual was heterozygous for the deletions, while seven were homozygous for each gene. The *CTSW* gene is encoding Cathepsin W, a cysteine proteinase that is selectively expressed in CD8^+^ T cells and natural killer cells, which are crucial for immune responses. The mutation specific to the Okazaki population leads to a frameshift at Q40X. Given the absence of an alternative start codon immediately following the frameshift site, it is probable that the Cathepsin W protein is nonexistent in Okazaki individuals, potentially influencing the immune characteristics of this group. Another candidate gene *ISG20L2* encodes an exoribonuclease that is activated by interferon and plays a significant role in immunity. Research by Rodríguez-Galán et al. (2023) has demonstrated that the lack of this gene can amplify the activation of T cells in cultured human cells. The deletion found in the Okazaki population affects the C-terminal region of the protein, removing sites essential for catalytic activity and likely leading to phenotypic differences.

### Population History of Japanese Macaques

The pattern of differentiation among six populations including Koshima individual implied a highly complex population history. The initial divergence among populations probably occurred around 150 to 200 kya between north-east and south-west groups. The estimated divergence timing within the Japanese archipelago is quite deep, considering that the split of RMI and RMC was estimated to have occurred around a similar time frame (Hernandez et al. 2007; Zhou et al. 2024), which overlaps with the penultimate glacial period. Furthermore, the trajectories of rCCR suggested that the process of divergence was not simple, and probably there have been multiple split and merge events following to their initial divergence. The gene flow between populations in the nuclear genomes was also inferred by Ito et al. (2021). In many species pairs, there were increases of rCCR ∼10 to 30 kya, when all population increased their within-population coalescent rates (a signature of population size decrease). If the shrink of population sizes is caused by habitat fragmentation, rCCR should decrease but the rates rather increased in our dataset, particularly between Tsubaki and Mihoh populations. The pattern therefore suggests that their habitats during the shrinkage of native range might have been interconnected or overlapped. The timing may correspond to the LGM, when the habitats of Japanese macaques might have been restricted to a few refugia along the southern coastal areas (Ito et al. 2021). Altogether, they may have expanded their range and start genetically diverging during interglacial periods with the isolation-by-distance mode and contracted the range and partly merge with the other populations during the glacial periods.

Clear genetic distinctions were identified between the Jigokudani–Hagachizaki and Minoh–Tsubaki–Okazaki populations. This differentiation echoes findings by Ito et al. (2021), which reported significant genetic divergence of populations from Shimokita—the northernmost point of Honshu—, Yamagata, and Gunma, compared with other groups. However, the Jigokudani and Hagachizaki populations are geographically located near the boundary of the north-east and south-west division. To ascertain whether the Jigokudani and Hagachizaki populations are part of the north-east group defined by Ito et al. (2021), we integrated our dataset with the ddRAD-seq data and constructed a Neighbor-Net graph, presented in supplementary fig. S4, Supplementary Material online. The integrated analysis confirmed the classification of the Jigokudani and Hagachizaki populations with the Shimokita, Yamagata, and Gunma populations, hence assigning them to the north-east group. Moreover, the Okazaki population aligned with the western Japanese Kansai area cluster, which is in agreement with the patterns observed in our whole-genome sequence analysis.

## Conclusion

The research delivers high-coverage whole-genome sequences for 64 Japanese macaques, shedding light on the genetic underpinnings of the species, with significant implications for both evolutionary biology and biomedical research. Our findings reveal well-differentiated local populations of Japanese macaques, each with a complex and dynamic evolutionary history involving multiple splits and merges over the past 500,000 years. The prolonged presence in the Japanese archipelago, coupled with their small effective population sizes, has resulted in each group harboring numerous unique genetic variants. Some of these variants have considerable effects on individual phenotypes and may underlie observed differences between populations. The genomic insights from this study pave the way for future research on these uniquely fascinating nonhuman primates.

## Materials and Methods

### Sample Preparation and Genome Sequencing

Whole blood cells for genomic DNA were obtained from 5 populations of macaques: the Okazaki (8 individuals), Jigokudani (17 individuals), Hagachizaki (15 individuals), Minoh (16 individuals), and Tsubaki (8 individuals) populations (Fig. 1; supplementary table S1, Supplementary Material online). They were housed at the Center for the Evolutionary Origins of Human Behavior (EHUB), Kyoto University, Inuyama, Aichi, Japan, in accordance with Guidelines for Care and Use of Nonhuman Primates (Ver. 3, 2010, Primate Research Institute, Kyoto University and Guidelines for the Captive Maintenance, Storage, and Utilization of Japanese Macaques 2018, NBRP).

Blood collection was conducted at the EHUB in accordance with the guidelines of the Laboratory Biosafety Manual, World Health Organization. Genomic DNA was isolated from 2 mL of peripheral blood with EDTA using a Qiagen Genomic DNA purification kit (Qiagen K. K., Tokyo, Japan). The isolated DNA samples were kept at −30 °C until use. The protocol was approved by the Animal Welfare and Animal Care Committee, the Center for the EHUB, Kyoto University (permission no. 2022-104, 2023-148, 2024-019).

Genome sequencing was performed using the DNBSEQ-G400 (MGI). Mate-pair (150 bp × 2) libraries were generated using the macaque genomic DNA. Paired-end libraries of 350 bp insert sizes were prepared. Library preparations and all DNBSEQ runs were performed as per the standard manufacturer’s protocols. Demultiplexing was performed by the DNBSEQ built-in software, and the raw data of each individual were subsequently retrieved by means of specific barcodes. All sequence reads have been deposited to the DDBJ database of the National Institute of Genetics, accession number DRA016173.

### Variant Calling and Filtering

In addition to the newly sequenced data, we also downloaded whole-genome sequence data for *M. fascicularis*, *M. mulatta*, *M. cyclopis*, and *M. fuscata* from public databases (supplementary table S2, Supplementary Material online) (Xue et al. 2016; Liu et al. 2018; Osada et al. 2021). Paired-end reads were aligned to the reference genome sequence (Mmul_10) using the Parabricks 4.0 fq2bam pipeline (Alser et al. 2020), which also sorted the reads and marked polymerase chain reaction duplicates. Initial genotyping was conducted with GATK HaplotypeCaller as implemented in Parabricks 4.0 (Van der Auwera and O'Connor 2020), followed by joint calling using GATK 4.4 GenomicsDBImport and GenotypeGVCFs. Hard filtering criteria for SNVs and indels were set as follows: for SNVs, “QD < 2.0, QUAL < 30.0, SQR > 3.0, FS > 60.0, MQ < 40.0, MQRankSum < -12.5, ReadPosRankSum < -8.0, ExcessHet > 30.0”; for indels, “QD < 2.0, QUAL < 30.0, FS > 200.0, ReadPosRankSum < -20.0, and ExcessHet > 30.0.” SNVs and indels were annotated using SNPEff 5.1 with the Mmul_10.105 annotation (Cingolani et al. 2012).

We further applied mappability and accessibility filters on the SNVs. The mappability score for each genomic position was calculated using GenMap software (Pockrandt et al. 2020). We specifically selected sites with a (30, 2)-mappability score of 1. This indicates that a 30-mer sequence starting from the filter-passed site is unique within the genome, even when allowing for two mismatches. To assess genome accessibility, we evaluated the read coverage at each site across all individuals. We determined the mode of the coverage distribution, and sites falling within the range of half to twice the mode value were classified as accessible, corresponding to 17.5- to 67.5-fold coverages per individual genome. Autosomes encompassed 1,930,114,258 sites in mappable and accessible regions. We also genotyped nonpseudoautosomal region (PAR) of X chromosomes. The non-PAR was assigned to chrX:2,364,962-151,635,290. Male and females were separately genotyped, and the variants were merged after the genotyping. The hardfiltering and mappability filtering were applied with the same criteria for autosomes. For the accessibility filter, we adjusted the coverage range calculated using autosomal data, assuming that the X chromosomes of males and females are haploid and diploid, respectively. We used BCFtools to identify regions of ROH (Narasimhan et al. 2016) Regions with Phred quality scores below 30 or shorter than 100 kb were filtered out.

### Haplotype Phasing

We first phased haplotypes with four trio groups, focusing exclusively on autosomal SNVs. Each trio sample was phased using WhatsHap 1.7, utilizing trio-based information (Martin et al. 2016). These phased haplotypes from the trios were then employed as a reference panel to help the statistical phasing of all Japanese macaque individuals. The statistical phasing was executed using SHAPEIT 5.1.1, conforming to the standard procedures outlined on the SHAPEIT5 website (Hofmeister et al. 2023). This included steps such as chunking, ligating, and phasing common and rare variants. Prior to being processed with SHAPEIT, each nontrio sample underwent initial phasing using WhatsHap 1.7. For the non-PAR of X chromosomal data, we only used SHAPEIT for haplotype phasing.

### Analysis of Population Structures

We utilized only biallelic SNVs for studying genetic structures. The filtered biallelic autosomal SNVs in the VCF file were converted into the PLINK 1.9 file format for population genetics analyses (Purcell et al. 2007). After format conversion, individuals with a kinship equal or higher than the third degree were excluded using PLINK 2.0’s “–king-cutoff 0.0884” option (Chang et al. 2015), resulting in the removal of 20 individuals (marked in supplementary table S1, Supplementary Material online). SNVs under strong linkage disequilibrium (LD) were pruned using PLINK 1.9 with “–indep-pairwise 50 5 0.5.” Subsequent PCA was also performed with PLINK 1.9. We used the LD-pruned dataset to run ADMIXTURE (Alexander et al. 2009). The genetic distance between individuals was estimated by calculating the genotype distance, where heterozygous sites for both individuals were assigned a value of 0.5. These data were then used with SplitTree4 for reconstructing a neighbor-joining tree and a Neighbor-Net graph (Huson and Bryant 2006). Admixtools was employed to estimate *f*_3_ and *f*_4_ statistics using the non-LD-pruned SNV dataset. (Patterson et al. 2012). *F*_ST_ for each SNV was estimated using Hudson’s method implemented in PLINK 2.0. Genome-wide *F*_ST_ was calculated by averaging the numerators and denominators, followed by taking the ratio (Bhatia et al. 2013). Confidence intervals were estimated using a jackknife block size of 25,000 SNVs, roughly corresponding to 5 cM in our dataset. For the analysis of LoF SNVs with high *F*_ST_, we considered variants that were successfully called in all unrelated Japanese macaque samples. Functional enrichment analysis of highly differentiated LoF mutations was performed using DAVID (Jiao et al. 2012). Enrichment in the three gene ontology categories (biological process, cellular component, and molecular function) was assessed using ENSEMBLE Gene IDs as queries, with a false discovery rate threshold of 0.05.

### Demographic Inference

The inference of demography was performed using MSMC2 (Schiffels and Wang 2020). In order to estimate effective population sizes of each population of Japanese macaque, for each population eight phased autosomal haplotypes from four unrelated individuals were used (supplementary table S9, Supplementary Material online). The population of Koshima was not analyzed because it contains only single individual. Mutation rate of 6.5 × 10^−9^ was used (Wang et al. 2020), assuming the generation time of 11 years (Itoigawa et al. 1992; Koyama et al. 1992).

### The Sequencing of Variants Within the TECR, CTSW, and ISG20L2 Regions

The sequencing was based on the Sanger technique and the BigDye Terminator V3.1 (Life Technologies, Carlsbad, CA, USA) was used. The primer sequences used for sequencing of *TECR*, *CTSW*, and *ISG20L2* regions are listed in supplementary table S10, Supplementary Material online. The sequencing protocol was as follows: 30 cycles of 10 s at 98 °C, 30 s at 55 °C, and 60 s at 72 °C, and finally the sequences were maintained at 4 °C. The DNA sequence was determined using the ABI3730x Genetic Analyzer (Thermo Fisher Scientific-Applied Biosystems, Foster City, CA, USA). The sequences obtained were read and aligned with the MEGA X (Knyaz et al. 2018).

### Analysis of ddRAD-seq Data

The ddRAD-seq data from 88 Japanese macaques, as reported by Ito et al. (2021), were retrieved from the NCBI sequence read archive database. A comprehensive list of the samples analyzed in this study can be found in supplementary table S11, Supplementary Material online. Note that these combined data were exclusively used for generating supplementary fig. S4, Supplementary Material online. We aligned the short reads to the rhesus macaque Mmul_10 reference genome using the Parabricks 4.0 software, consistent with the procedures applied to whole-genome sequencing data. SNVs were genotyped employing the Stacks software pipeline (Catchen et al. 2013), and the resulting variant data were integrated with the whole-genome sequence data utilizing the BCFtools program. Prior to the integration, the orientation of reference and alternative alleles, as outputted by Stacks, was normalized using BCFtools. We included only those autosomal sites where more than 80% of the samples were successfully genotyped, assuring that these sites were variant sites in both datasets. The combined dataset was used to construct a Neighbor-Net graph, applying the method consistent with that used for the whole-genome sequence data analysis.

## Supplementary Material

evaf001_Supplementary_Data

## Data Availability

Raw sequencing reads are available in the DDBJ database of the National Institute of Genetics, accession number DRA016173.
