# Correction to: Population Genomics of Japanese Macaques (*Macaca fuscata*): Insights Into Deep Population Divergence and Multiple Merging Histories

**DOI:** 10.1093/gbe/evaf087

**Published:** 2025-05-16

This is a correction to: Atsunori Higashino, Katsuki Nakamura, Naoki Osada, Population Genomics of Japanese Macaques (*Macaca fuscata*): Insights Into Deep Population Divergence and Multiple Merging Histories, *Genome Biology and Evolution*, Volume 17, Issue 1, January 2025, https://doi.org/10.1093/gbe/evaf001

In the originally published version of the manuscript, the supplementary data file had errors in the accession number tags in Supplementary Table S1. The file is now replaced in the article with emendations made to these errors.

