# Supplementary material for: Population Genomics of Japanese Macaques (Macaca fuscata): Insights Into Deep Population Divergence and Multiple Merging Histories
Source: Genome Biol Evol. 2025 Jan 7;17(1):evaf001. doi: 10.1093/gbe/evaf001 (PMC11735745; doi:10.1093/gbe/evaf001)
Supplement: evaf001_Supplementary_Data [file evaf001_supplementary_data.zip › Supplementary_Figures.pdf]

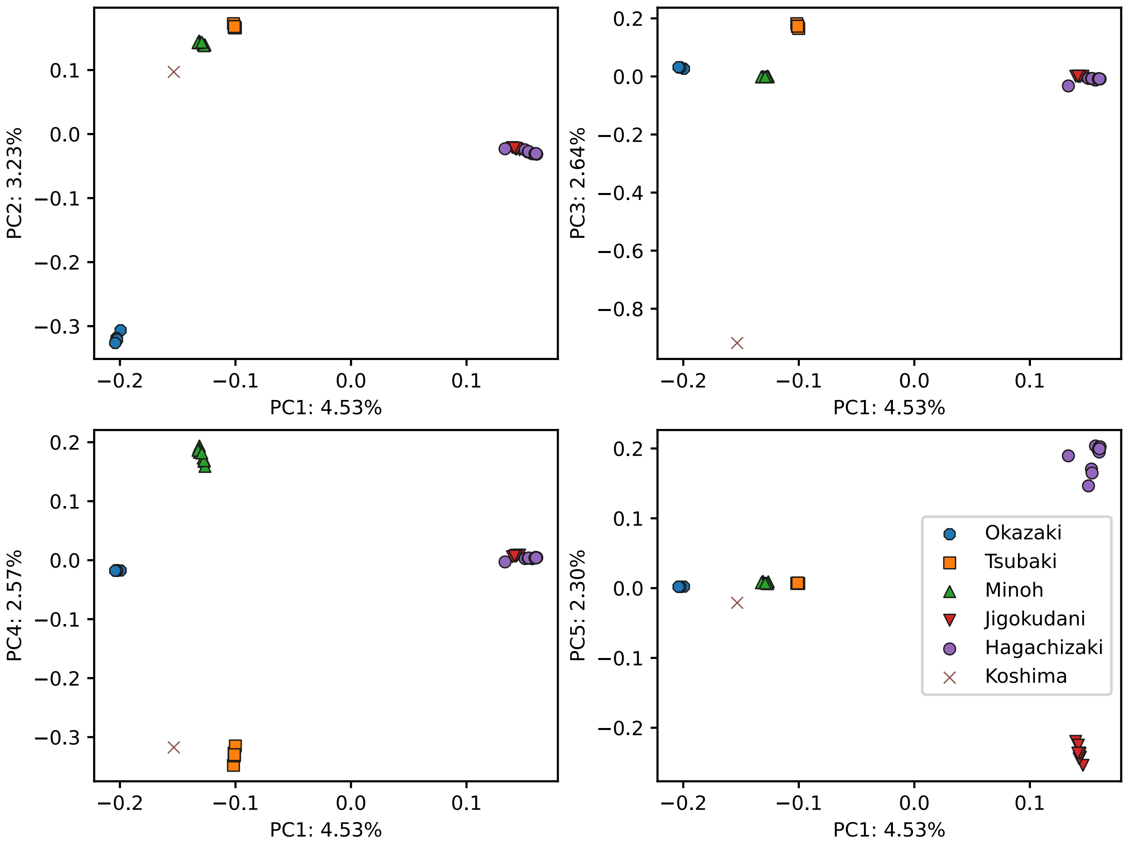

Supplementary Figure S1

Principal Component Analysis (PCA) results. In all four panels, the *x*-axes represent PC1 scores, while PC2, PC3, PC4, and PC5 scores are displayed in the *y*-axes of their respective panels.

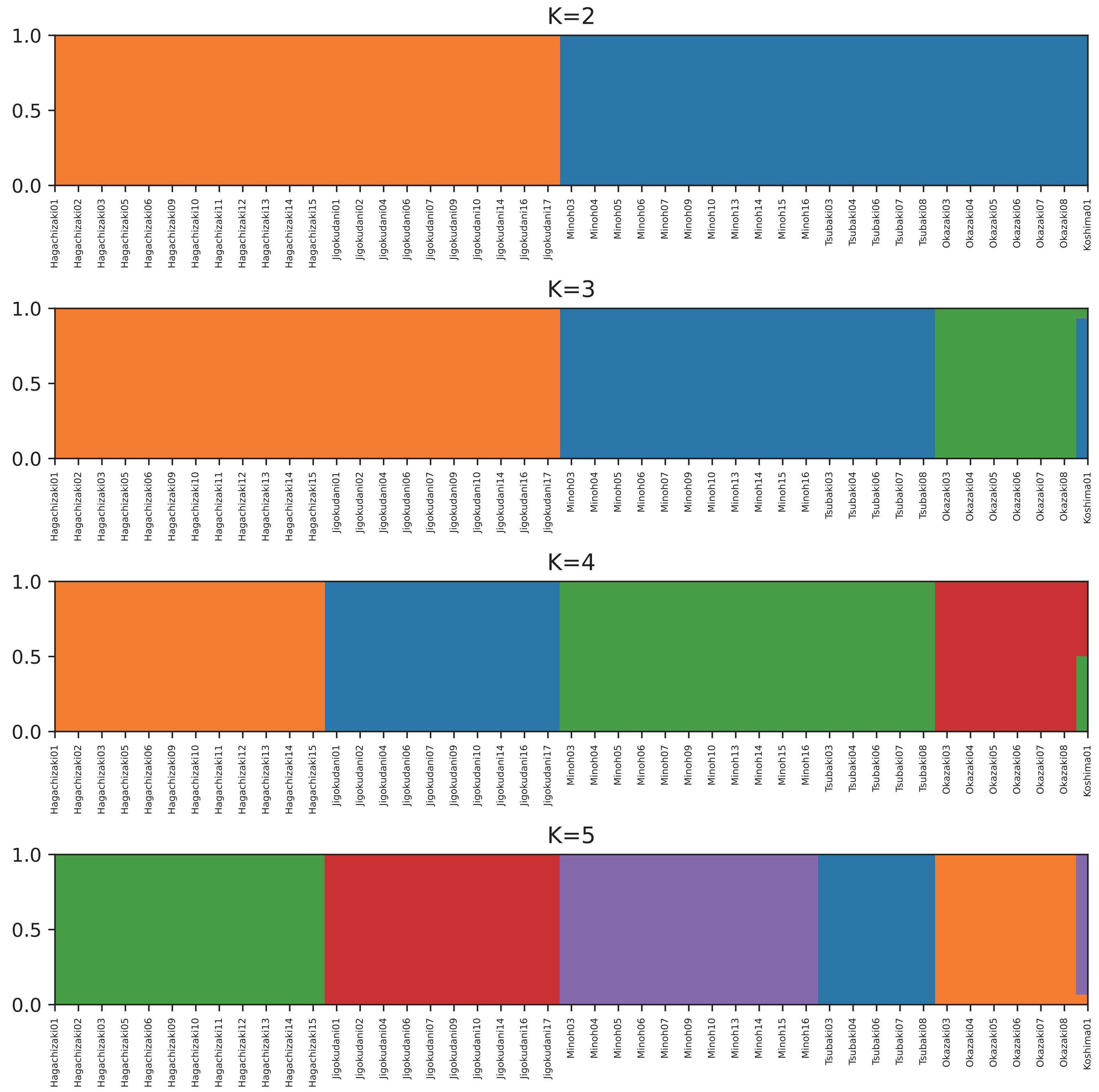

Supplementary Figure S2

Results of Admixture for the Japanese macaque samples, shown for K values ranging from 2 to 5.

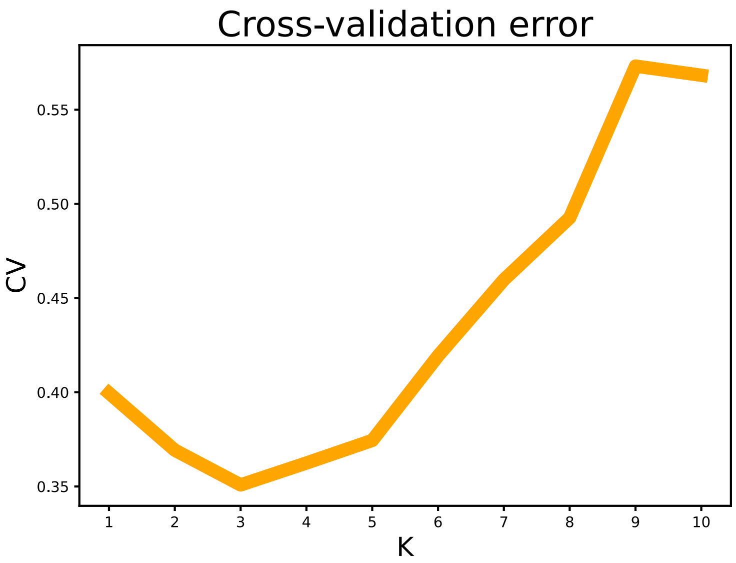

Supplementary Figure S3

Cross-validation errors of Admixture, shown for K values ranging from 2 to 10.

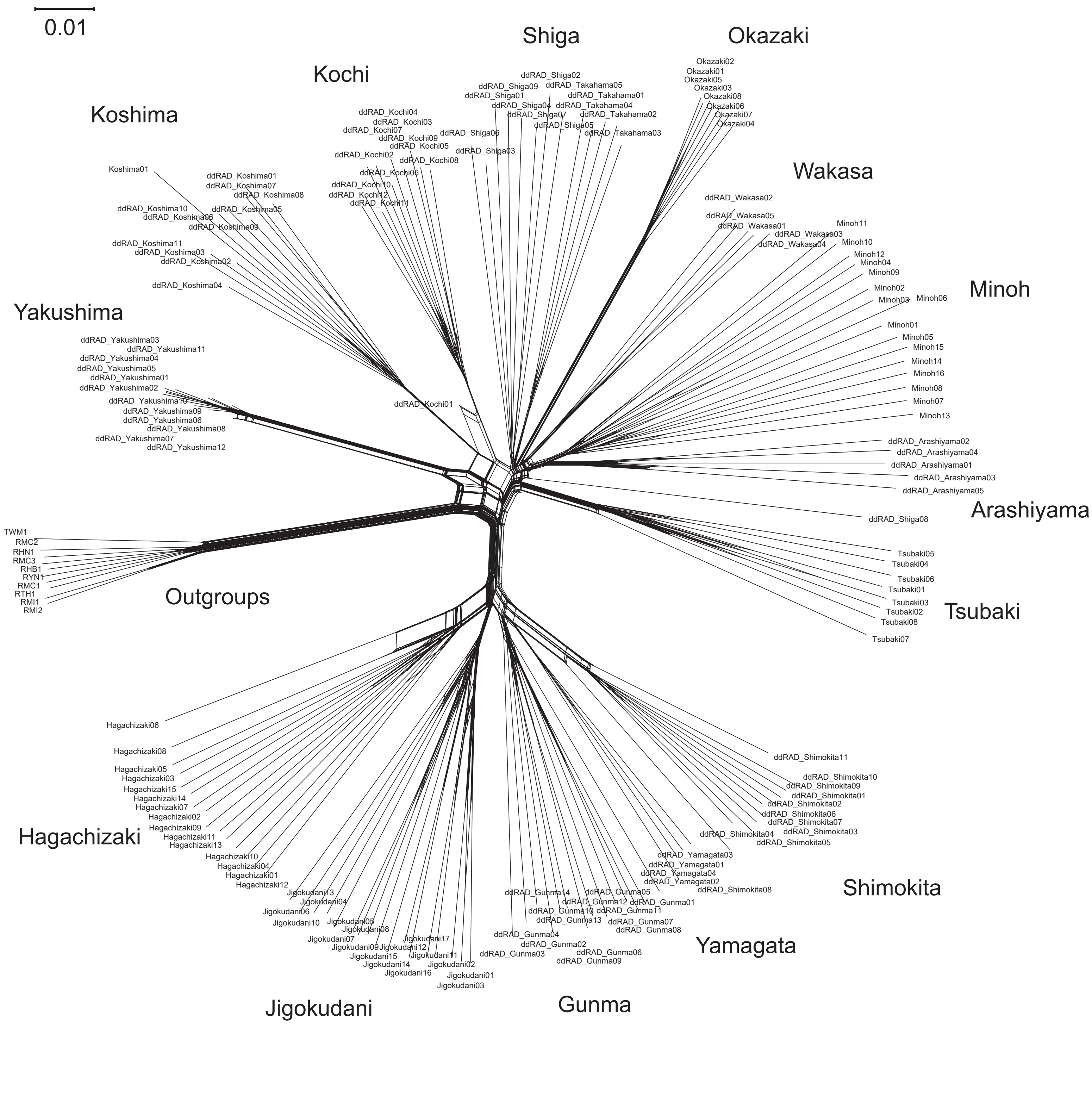

Supplementary Figure S4

Neighbor-Net graph including the whole-genome sequence dataset and ddRAD-seq data by Ito et al. (2021).
